# A compendium of temperature and salinity profiles and discrete nutrients from selected NOAA programs in Alaska

**DOI:** 10.1038/s41597-025-06342-5

**Published:** 2025-12-12

**Authors:** Calvin W. Mordy, Noel A. Pelland, Shaun W. Bell, Wei Cheng, Jeanette C. Gann, Albert J. Hermann, Caitlyn R. McFarland, Jens M. Nielsen, Phyllis J. Stabeno, Margaret E. Sullivan, Eric S. Wisegarver

**Affiliations:** 1 Cooperative Institute for Climate, Ocean, and Ecosystem Studies, University of Washington, 3737 Brooklyn Ave NE, Seattle, Washington 98105 USA; 2 NOAA Pacific Marine Environmental Laboratory, 7600 Sand Point Way N.E., Seattle, Washington 98115 USA; 3 NOAA Alaska Fisheries Science Center, 7600 Sand Point Way N.E., Seattle, Washington 98115 USA; 4 NOAA Alaska Fisheries Science Center, 17109 Pt. Lena Loop Road, Juneau, Alaska 99801 USA

**Keywords:** Physical oceanography, Marine chemistry

## Abstract

To better understand ecosystem dynamics in the Gulf of Alaska, Bering Sea, and Chukchi Sea, researchers at the U.S. National Oceanic and Atmospheric Administration’s (NOAA’s) Pacific Marine Environmental Laboratory and Alaska Fisheries Science Center have been conducting hydrographic and biological surveys in Alaska waters for decades. This article describes a new data compendium (“ACOD”) that assembles data sets from select NOAA programs into a single-point-of-access quality-controlled product. Included are 29717 vertical profiles of temperature and salinity (1974–2021), and 7016 profiles of dissolved inorganic macronutrients (nitrate, nitrite, ammonium, orthosilicic acid, and phosphate) at discrete depths (2001–2021). This value-added product includes systematic quality control of metadata, salinity, and nutrient data – in addition to creating a single point of access for data from 495 cruises across a nearly 50-year time period. ACOD files are archived at the Dryad Research Data Repository and will include annual or biennial updates. File types include netCDF (profiles and nutrients) and csv (nutrients), and a table with metadata from each cruise.

## Background & Summary

### Managing change in Alaska marine ecosystems

Alaska waters are home to five regionally managed Large Marine Ecosystems (LMEs^1,2^) including the Gulf of Alaska, Aleutian Islands, and the Bering, Chukchi, and Beaufort Seas. These highly productive regional ecosystems contain vast natural resources that support commercial and subsistence harvest. The Gulf of Alaska (GOA) and eastern Bering Sea (EBS) support some of the largest commercial fisheries in the U.S.^3^, and the Pacific Arctic Region (Bering, Chukchi, and Beaufort Seas) is one of the most productive seasonal marine ecosystems in the world^4–6^, supporting vast numbers of marine mammals and birds. These ecosystems are under stress from general warming and episodic events such as the marine heatwave (“The Blob”) that occurred in 2014–16 in the GOA^7–9^. In the Pacific Arctic, climate forcing is reducing seasonal ice and increasing ocean temperatures^5,10,11^. Impacts of reduced sea ice include: changes in primary productivity^12,13^ and in phytoplankton and zooplankton community structure^14,15^; bird die-offs^16^ and changes in the migration patterns of fish, birds and whales^17–19^; and impacts on commercial fisheries^20,21^ and communities reliant on these marine resources^18^.

The U.S. National Oceanic and Atmospheric Administration (NOAA) serves as a steward of marine fisheries in Alaska waters. NOAA’s observational network includes moorings, annual fish and marine mammal surveys, meteorological and oceanographic studies, and broader surveys of marine ecosystems in Alaska waters. These data, along with observations and modeling from private, university, state, and federal researchers and agencies, are compiled into biological and physical indicators and utilized in Ecosystem Status Reports, Stock Assessments, and Ecosystem and Socioeconomic Profiles that inform the North Pacific Fishery Management Council and their ecosystem-based approach to management^22–24^.

### Compilation of hydrographic data in Alaska waters; a new value-added data product

Amongst the vast array of NOAA mooring and survey data in Alaska, the Pacific Marine Environmental Laboratory (PMEL) and the Alaska Fisheries Science Center (AFSC) have collections of hydrographic data in Alaska waters that span decades. This article describes a new compendium of observational data from these NOAA laboratories, titled as the Alaska Compendium of Ocean Profile Data (“ACOD”) and intended for public distribution and use in oceanographic, ecosystem, and climate research. The data included in this effort are focused on three major modes of data collection by NOAA in Alaska waters. Firstly, the compendium includes sampling by the Ecosystems and Fisheries-Oceanography Coordinated Investigations (EcoFOCI) program, a multi-decade scientific initiative composed of scientists at both PMEL and AFSC. Secondly is the inclusion of data from the Ecosystem Monitoring, and Assessment (EMA) program led by AFSC with field work and data processing supported by PMEL/EcoFOCI. Finally, an extensive record of hydrographic data collected between the founding of PMEL in the early 1970s and the beginning of EcoFOCI in 1984 is also included. These programs are described in more detail below.

The intent of ACOD is to assemble these disparate data sets into a single-point-of-access data product that includes additional quality control of the data and metadata. As such, this product complements and builds upon existing cruise data from these programs that have been publicly archived in a variety of locations.

This value-added compilation focuses on basic hydrographic variables collected from shipboard profiling instruments and bottle samples including ocean temperature, salinity, dissolved nutrients (phosphate, orthosilicic acid [hereafter silicic acid], nitrate, nitrite, ammonium) and pressure. ACOD is archived on the Dryad open-data publishing platform^25^ for research which facilitates long-term preservation and version control. This compendium represents most of the temperature and salinity profiles collected by PMEL in Alaska waters, but does not include other parameters from the Conductivity, Temperature, and Depth (CTD) profiler (e.g., oxygen, chlorophyll), moored records, or other NOAA hydrographic data sets such as those from AFSC’s Bottom Trawl Survey^26^. Between 2008 and 2019, PMEL attached ruggedized CTDs to the net hauls during AFSC bottom trawl surveys to obtain gridded data sets of temperature and salinity measurements on the eastern Bering Sea shelf^27^. AFSC began leading these measurements with different sensors in 2021. These data are being assembled and quality controlled at PMEL and may be added to the compendium in future updates. ACOD focuses on NOAA-related data only and does not include US academic or international sampling that is not in partnership with NOAA in this region (e.g.^28–31^).

### A brief history of PMEL, EcoFOCI, and EMA

The history of NOAA-related observational oceanography in Alaska is lengthy, and a full description is beyond the scope of this work. We only briefly summarize the important institutions that contributed to ACOD here; for more comprehensive accounts, readers are referred to several recent works that review the history of EcoFOCI and related programs^23,32,33^. PMEL was established in 1973 and soon after began environmental studies in Alaska waters that focused on the physical dynamics of the GOA, EBS and Chukchi Sea. These studies included time series data from moorings and vertical CTD profiles of the water column. These older (through the early 1990s) data sets were usually archived at the NOAA National Oceanographic Data Center (NODC), now reorganized into the NOAA National Centers for Environmental Information (NCEI).

In 1984 a new program was established between oceanographers at PMEL and biologists at AFSC that is now known as EcoFOCI^23,32^. Long-term mooring sites were established^33^ and regular hydrographic and biological surveys were undertaken to monitor the seasonal and interannual dynamics of these ecosystems. For example, in the EBS, EcoFOCI conducts spring and late summer/early fall mooring cruises that incorporate hydrographic sampling along portions of the 70-m isobath^34,35^. In the Chukchi Sea, EcoFOCI began annual mooring and hydrographic surveys in 2010 with a variety of partners^36^. In addition, EcoFOCI has worked with private, university, state and other NOAA and federal partners in a variety of specialized research projects and programs (e.g., Aleutian Archipelago study^37^; Bering Sea study^38^; Arctic Integrated Ecosystem Research Program (AIERP)^39^; GOA Research program^40^). Since the founding of EcoFOCI, CTD data have been quality controlled and archived in different repositories depending on requirements of the funding agency. A search through NOAA’s World Ocean Database^41^ found that at least 64% of the cruises had at least one type of archival data in NCEI. Accession numbers and links to these data are included in the data product. While PMEL CTD data extend back to 1974, PMEL began processing nutrient samples in 2001. As with the recent CTD data, nutrient data were archived in different repositories per the requirements of the funding source.

AFSC’s EMA program conducts fisheries research in the GOA, Bering Sea, and Chukchi Sea. Beginning in 1999, the North Pacific Anadromous Fish Commission began an internationally coordinated research program on salmon in the Bering Sea designated as the Bering-Aleutian Salmon International Survey (BASIS^42^). As part of this program, AFSC began to survey fish populations over the southeastern Bering Sea shelf along a regularly spaced sampling grid (0.5° latitude × 1° longitude), although not all stations were sampled each year. These surveys were extended into the northern EBS and the Chukchi Sea through several external funding sources including the Bureau of Ocean Energy Management (BOEM) in support of the Arctic Ecosystem Integrated Survey (EIS^43^), and the North Pacific Research Board AIERP. Since 2016, sampling in the southeast Bering Sea is biennial during even years while sampling in the northern Bering Sea continues to be an annual survey. CTD profile data were processed and quality controlled by EMA from 1999 to 2011, and by PMEL thereafter. Likewise, nutrient samples were processed by two different laboratories; the Marine Chemistry Laboratory at the University of Washington (UW-MCL; 2003–2011) and PMEL (2012-present).

## Methods

### Sampling design overview

**Fig. 1:**
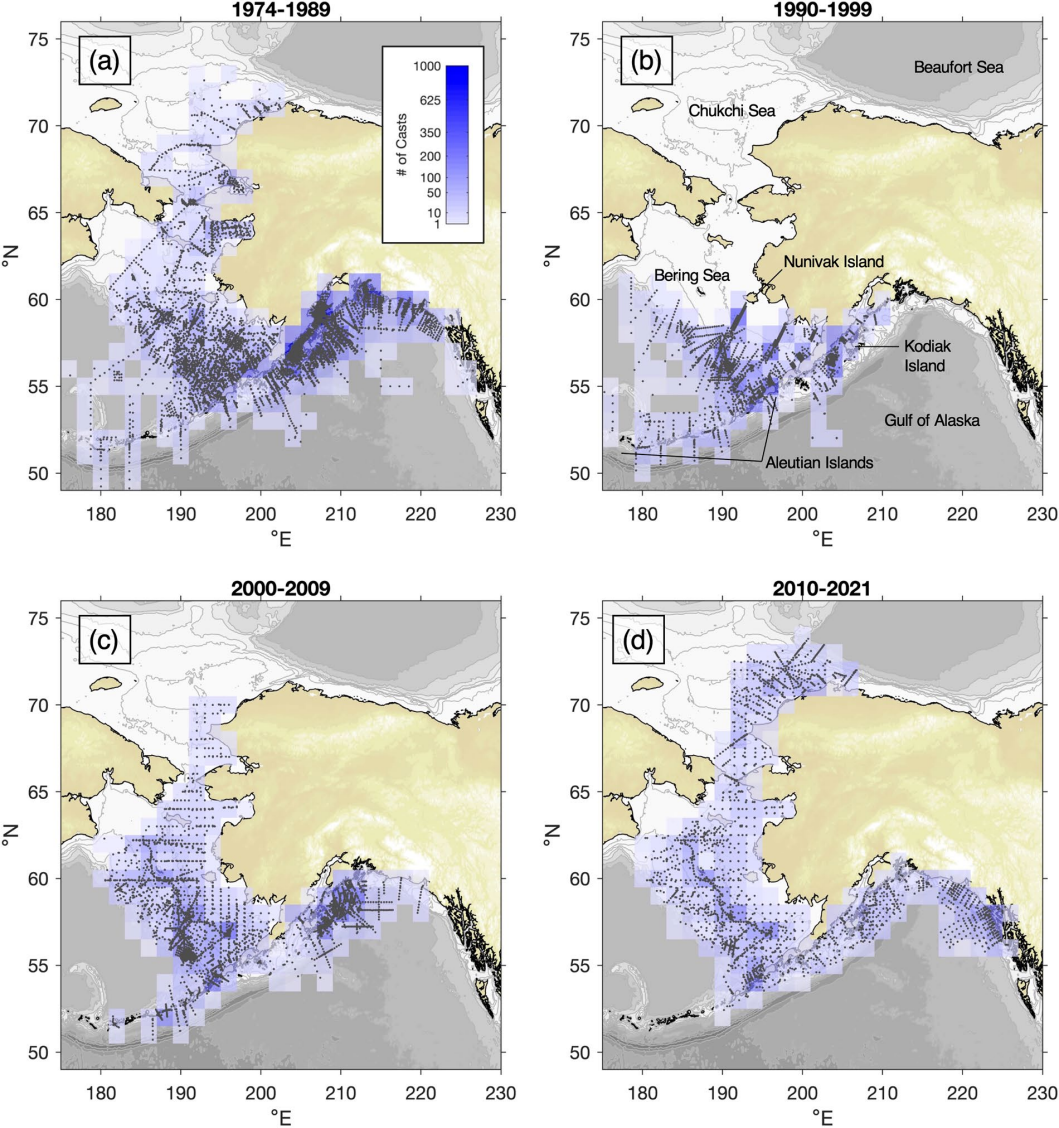
Density of CTD profile data included in ACOD v1.0, through four eras of sampling: (**a**) 1974–1989, (**b**) 1990–1999, (**c**) 2000–2009, and (**d**) 2010–2021. Black dots represent individual cast locations, whereas background shading shows cast density in 1° latitude × 2° longitude spatial bins during each era. The color scale for cast density is shown in panel (**a**).

**Fig. 2:**
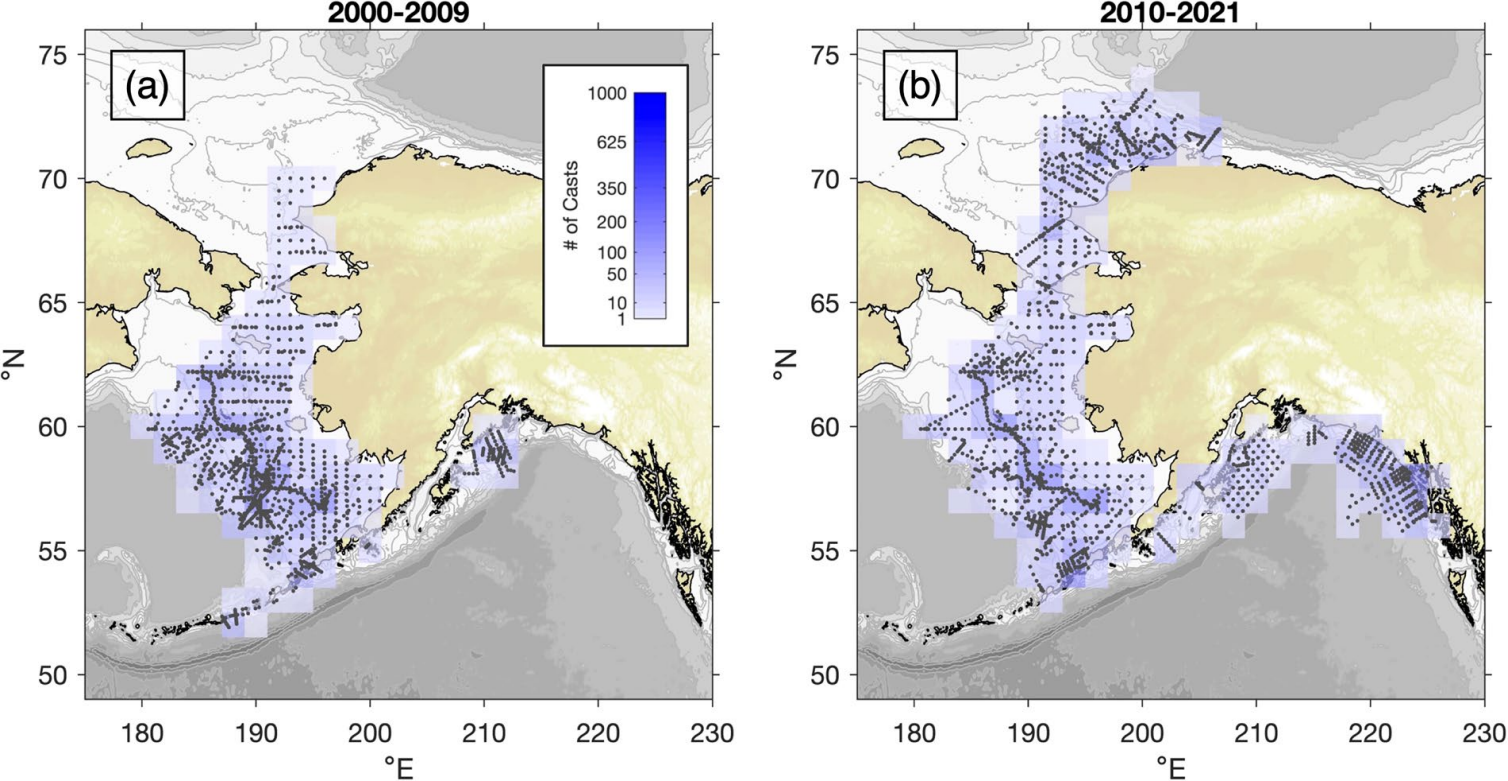
Density of profiles with nutrient data included in ACOD v1.0, plotted by era as in Fig. 1, for (**a**) 2000–2009, and (**b**) 2010–2021. The earliest included nutrient data were collected in 2001.

These data sets cover a region of >3 million km^2^, ranging from the southern edge of the GOA to the Arctic Ocean, and from Alaska westward to Asia, with a focus on the eastern continental shelves. CTD data ultimately used in the compendium were drawn from the PMEL and AFSC digital archives, and included 495 cruises spanning July 1974 - September 2021. The sampling contained in this collection is equivalent to more than 16 continuous years at sea, with 7 + million meters of total profile extent. The evolution of the observational program is shown in Fig. 1. Prior to 1990, observations extended over four of Alaska’s LMEs with the most intense sampling in southeastern Bering Sea and in the vicinity of Kodiak Island. In the 1990s, sampling did not extend north of Nunivak Island with research focused in the southeastern Bering Sea and the northern GOA. In the 2000s, research returned to the northern Bering and Chukchi Seas, and in the last decade observations have expanded from southeast Alaska to the Beaufort Sea with less sampling along the Aleutian Islands. Since 2003, nutrient samples have been collected on virtually all of these cruises with cast locations shown in Fig. 2, and the earliest nutrient data are from 2001.

**Fig. 3:**
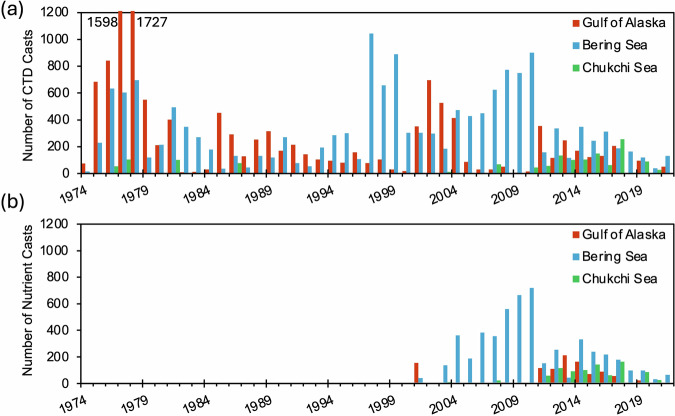
Histograms showing (**a**) the number of CTD casts and (**b**) the number of casts for nutrients per year in the Gulf of Alaska (red) Bering Sea (blue) and Chukchi Sea (green).

The histograms in Fig. 3 show the annual number of CTD casts and nutrient casts in each region. To a large extent, variability in the regional and temporal coverage reflects specific research projects and programs. For example, the large number of casts in the GOA and Bering Sea during the 1970s were associated with the Outer Continental Shelf Environmental Assessment Program (OCSEAP^44,45^), and intense sampling in the Bering Sea from 2007–2010 was associated with the Bering Sea Ecosystem Study (BEST^38^).

### Instrumentation, sampling and analytical protocols

#### Instrumentation

Measurements of ocean temperature and salinity (determined from conductivity) were collected using a profiling CTD which was developed around the time PMEL was founded^46^. Over the 47 years of data presented in this compendium, a variety of CTD instruments were used to gather data. Prior to 1988, Plessey instruments (e.g., model 9040) were common for OCSEAP sampling^47^. Since approximately 1988, the instrument of choice has been the Sea-Bird Scientific (SBE) 911plus system, although on some earlier EMA cruises (prior to 2018), an SBE25 was used.

While some information can be found in the literature, protocols for sampling and data processing were not always evident for data collected prior to the founding of EcoFOCI. In the formative years of the CTD (1970s), data quality in highly stratified waters was often compromised due to rapid descent of the CTD through a sharp thermocline, a feature that develops in the Bering Sea during summer, and can reach ~10 °C m^−1^. For EcoFOCI (since 1985), the CTD was lowered and held at ~10 m to ensure instrument stability, raised to the near surface (usually 2–4 m), and then the cast was initiated with data being recorded during the downcast. The CTD was lowered at a descent rate of 15 m min^–1^ to a depth of ~35 m, and 30 m min^–1^ below that, although descent rates can vary with sea state. In deeper water (below ~400 m), the descent rate was often larger (~60 m min^–1^).

Since 1987, common post-processing procedures include, but are not limited to, the following: filtering high frequency noise, removing ascending loops, binning to a standard resolution of 1 dbar, calculating derived parameters, despiking, compensating for pumping/sampling offsets, compensating for thermal lags, establishing instrument integrity/validity, and applying calibration/characterization offsets. Instrument calibrations were provided by the manufacturer.

Because the CTD casts usually begin 2–4 m below the surface the uppermost value was extended upward to the surface. (This was the methodology for data collected after 1985.) If data recording started at deeper depths (>~8 m), the surface was denoted as missing data. If there was a single spurious value in the data record, this data point was modified by linear interpolation. If there were multiple, consecutive spurious values, these were marked as missing data. Before the advent of dual temperature and salinity sensors, salinity calibration samples were collected on each or every other up-cast and analyzed using a laboratory salinometer. After the introduction of dual sensors, salinity samples continued to be collected on every fourth or fifth cast; this has recently been discontinued unless the conductivity channels begin to drift apart.

#### Vertical profiles of temperature and salinity

The vertical coordinate for the compendium data is pressure in units of dbar. Within the compendium, the 1 dbar resolution is retained to a level of 1500 dbar. For casts extending deeper than this, the archival data were linearly interpolated to a 5 dbar grid, to a maximum of 5000 dbar. If the archival data had dual CTD sensors, the best quality data were designated as the “primary temperature” and “primary salinity” channels and incorporated in this compendium.

All temperature data prior to August 1995 and two cruises on the R/V *Alpha Helix* (hx196 [1996] and hx209 [1998]) were recorded in the International Practical Temperature Standard 1968 (IPTS-68). For the compendium, these were linearly converted to the International Temperature Scale 1990 (ITS-90) standard following Saunders *et al*.^48^, as implemented in the International Thermodynamic Equation of Seawater–2010 manual^49^. All other cruises recorded *in situ* temperature under the ITS-90 standard.

Salinity data in the compendium are presented in the units in which they were originally recorded. Prior to August 1995 (cruise su9503a), salinity was recorded in units of parts per thousand (PPT). Data from August 1995 onward were recorded as Practical Salinity (Practical Salinity Scale 1978; PSS-78^50^). The salinity value in PPT will differ from PSS-78 by an amount that will depend on the original salinity algorithm that was used, how each instrument was field calibrated, and the temperature, salinity, and depth of each sample^51^. Multiple algorithms were in popular use prior to PSS-78 for converting field measurements of conductivity and temperature to salinity. Information regarding historical salinity algorithms and field calibration data were not consistently available for cruises reporting in PPT, hence, conversion of these profiles to PSS-78 was not attempted. For typical values of T and S in this region, the differences between PPT and PSS-78 are likely to be in the range of 0.01–0.05^51^. Conversion of values in PSS-78 to absolute salinity S_A_ (g/kg) can be accomplished using widely available algorithms in the Gibbs Seawater Oceanographic Toolbox^52^ along with sample latitude, longitude, and pressure information.

#### Nutrient sampling and analysis

The compendium includes dissolved inorganic nutrient data (phosphate, silicic acid, nitrate, nitrite and ammonium) from PMEL/EcoFOCI and EMA. Samples were collected at discrete depths from Niskin bottles attached to the CTD rosette and were measured using autoanalyzers with segmented flow. Protocols utilized by PMEL and UW-MCL were comparable and included calibration of labware, preparation of primary and secondary standards, and corrections for blanks, refractive index and carryover. Ammonium was analyzed on most, but not all cruises.

PMEL typically sampled at 10-m depth intervals from the surface to 50 m, and at increasing intervals in deeper water. In waters < 200 m depth, the deepest sample was usually collected within 12 m of the bottom. To reduce analytical noise, samples were syringe-filtered using 0.45 µm cellulose acetate membranes. Sampling gear was rinsed 3 times before sample collection. Samples were either frozen for later analysis at PMEL (typical for mooring cruises) or analyzed at sea (typical for larger programs). All sample bottles had sufficient headspace to allow for thermal expansion and were stored upright. This was especially important for frozen samples to ensure that brine was not extruded through the cap. Freezing generally reduces the precision of nutrient analysis, but does not bias the results so long as consideration is given to polymerization of reactive silicic acid^53–55^ (discussed below). This is addressed by re-running the samples for silicic acid 24–48 hours after thawing at room temperature, and only using silicic acid data from the second analytical run.

PMEL used a custom-built autoanalyzer (using components from Alpkem, Perstorp, and Technicon) until 2015, and thereafter used Seal AA3 and A500 autoanalyzers. Sample analysis followed protocols from the World Ocean Circulation Experiment (WOCE) program^56^ which was updated for the Global Ocean Ship-based Hydrographic Investigations Program (GO-SHIP)^57,58^. Standards were prepared for each cruise, and standard concentrations were occasionally validated against commercial standards from Ocean Scientific International Ltd. Detailed chemical protocols appear below.

EMA sampling in the Bering Sea from 2003 - 2011 only collected discrete nutrient samples from three to five depths, and the deepest sample was frequently >12 m off the bottom. Samples were stored frozen without filtration and sent to UW-MCL for analysis following the same WOCE protocol cited above. After 2011, nutrient sampling (i.e., sampling depths, filtration) followed the PMEL protocols, and sample analysis was conducted by PMEL.

Nutrient analytical protocols used by PMEL and UW-MCL were similar. Dissolved inorganic phosphate was measured by adding acidified ammonium molybdate to the sample stream followed by the addition of a reducing agent (dihydrazine sulfate or ascorbic acid) which forms a phospho-molybdenum blue complex. The mixture was heated to complete the reaction, and the absorbance was measured at 820 or 880 nm. Dissolved silicic acid was measured by adding acidified ammonium molybdate and tartaric acid (to reduce interference with phosphate) to the sample stream followed by the addition of a reducing agent (stannous chloride or ascorbic acid) which forms a silico-molybdenum blue complex. The absorbance was measured at 820 or 880 nm. Dissolved nitrite was determined by complexing nitrite with sulfanilamide and N-1-naphthylethylenediamine dihydrochloride to form a red azo dye and measuring the absorbance at 520 nm. Dissolved nitrate was determined by mixing the sample stream with an imidazole buffer and passing this mixture through a cadmium column which reduces nitrate to nitrite. Total nitrite (reduced nitrate plus nitrite) was measured as above, and nitrate was determined by difference. Ammonium was measured using two different methods. Prior to 2015, colorimetric detection was used for all parameters including a modification of the Mantoura and Woodward^59^ indophenol blue method for analysis of ammonium. In this method, the sample stream is treated with alkaline phenol and hypochlorite, and catalyzed with sodium nitroprusside under heat to form an indophenol blue complex which is measured at 640 nm. In 2015, PMEL switched to fluorometric detection of ammonium using an ortho-phthaldialdehyde (OPA) reagent^60^. In the fluorometric method, the sample is treated with OPA, sodium sulfite, and borate buffer reagent, then heated to 75 °C. The fluorescence was measured at 460 nm after excitation at 370 nm.

## Data Records

### Overview

The data record^25^ consists of five files which are stored in the Dryad Research Data Repository (10.5061/dryad.gf1vhhn0t). In these files, each cruise is assigned a unique number (1 to 495) that is separate from the cruise name (e.g., dy1504), and each cast within the compendium is assigned a unique profile number (1 to 29717) that is independent from the cast number within a cruise. The three netCDF-4 files in this data product adhere to the Climate and Forecast Metadata Conventions version 1.8^61,62^.

1. ACOD_CTD_v1.0.nc is a netCDF-4 file containing profile data and metadata of temperature and salinity. Missing data are indicated by 1.e35.
2. ACOD_CTD-250_v1.0.nc is a truncated version of File (**1**) which includes only the top 250 dbar of data.
3. ACOD_NUT_v1.0.nc is a netCDF-4 format file containing nutrient data at discrete depths along with corresponding temperature and salinity from **(1)**, metadata, some derived quantities (e.g., total dissolved inorganic nitrogen [DIN]), and quality flags. Missing data are indicated by 1.e35.
4. ACOD_NUT_v1.0.csv is a version of File **(3)** that is stored in a comma-separated-value format, and missing data are indicated by “NA”.
5. ACOD_CRUISE_v1.0.csv is a comma-separated-value table containing cruise metadata.

### Temperature and salinity files

The primary contents of Files **(1)** and **(2)** consist of two-dimensional (pressure and cast/profile) arrays, which include temperature (variable name “Temperature”) and several parameters of salinity that are discussed below. Each row (n = 29717) of these arrays corresponds to the assigned profile number described by the variable “PROFILE”. Columns (n = 2201 for (**1**), or n = 251 for (**2**)) correspond to the pressure grid, which is described by the variable “PRESSURE”.

Metadata for each profile in Files **(1)** and **(2)** includes the following parameters. “CRUISE_NUMBER” is the uniquely assigned cruise number (1 to 495) that corresponds to cruise numbers listed in File **(5)** which is described below. “CRUISE_NAME” is the cruise name (e.g., dy1504) associated with each profile. “CAST” is the profile number within each cruise. “TIME” is the profile time (UTC) in days since 1 January 1900. “LATITUDE” and “LONGITUDE” are the profile latitude and longitude in degrees north and east, respectively. “BOTTOM_DEPTH” is the profile bottom depth in meters as measured in the field, available on ~ 96% of all profiles. All data fall into one of three geographic regions: Chukchi Sea, Bering Sea, or Gulf of Alaska and are indicated by the variable name “REGION” and the strings “Chukchi Sea”, “Bering Sea”, and “Gulf of Alaska”.

During the sampling period of this compendium, salinity was recorded on two different scales; parts per thousand (PPT) for data prior to August 1995, and practical salinity (PSS-78) for data after 1995. Therefore, salinity data are stored in separate variables depending on the scale in which they were originally recorded: “Salinity_PPT” and “Salinity_PSS”. There are also variables in which questionable salinity points have been deleted using criteria that are described below in the Technical Validation section (“Salinity_PPT_with_QC_applied” and “Salinity_PSS_with_QC_applied”).

The contents of File **(2)** are identical to File **(1)** with the exception that the data set is truncated at 250 dbar. File **(2)** is therefore smaller and may be easier to work with for users interested in near-surface data or on continental shelves.

### Nutrient files

The nutrient data in Files **(3)** and **(4)** include nitrate (variable “NO3”), nitrite (“NO2”), ammonium (“NH4”), phosphate (“PO4”), and silicic acid (“H4SiO4”) in units of micromole per liter (µM). Compared to per-mass units that are typically reported for the deep sea, units per volume are not greatly influenced by pressure in shallower water and are preferred by biochemists and biologists that use these data on the shallow Alaska shelves (e.g., integrating rates of drawdown into µmol m^−2^ d^−1^). During this process it was discovered that several cruises had previously been archived in per-mass units, or had incorrect unit labels. We incorporated original (prior to archiving) data records in units of µM in order to standardize these cruises with the units of the remaining dataset.

Because nutrient data were collected at specific depths, they are sparser and more irregular than CTD data. Nutrient data are stored in a single dimension, that is, nutrient data from all profiles are concatenated into a single column vector, rather than interpolated to a regular pressure grid as in the CTD data. File **(4)** (.csv) is a tabular, comma-separated-value file that includes the same contents as File **(3)**, minus the variable “SAMPLE”, which is an arbitrary indexing variable for the sample dimension (i.e., a row number). File (**4**) has two header rows indicating variable names and units.

In both the netCDF and csv nutrient files sample metadata include pressure “PRESSURE”, profile time (“TIME”), location (“LONGITUDE”, “LATITUDE”), bottom depth (“BOTTOM_DEPTH”), region (“REGION”), cruise number and name (“CRUISE_NUMBER”, “CRUISE_NAME”), and cast number (“CAST”). Where available, the Niskin bottle number from which the sample was drawn is included (“BOTTLE_NUMBER”). For some older cruises, measurements extracted from multiple bottles tripped at the same depth were assigned an artificial, concatenated bottle number in archival records (e.g., “45” for Niskins 4 and 5 tripped at the same depth). These artificial bottle numbers were removed in ACOD where they could be identified. Specifically, bottle numbers >36 – a typical maximum rosette size on most vessels – or >12 for cruises on the NOAA ships *Oscar Dyson* or *Miller Freeman* were set as missing data. Bottle numbers for EMA nutrient sampling prior to 2012 are also not included in the present version of ACOD. Bottle number is populated for ~90% of nutrient samples.

Two derived nutrient variables are included that represent total dissolved inorganic nitrogen: “DIN” and “DIN0”. The “DIN” values are equal to the sum of the NO3, NO2, and NH4 values for the same sample. If any of the three are not populated, the DIN variable is not populated. The “DIN0” variable is similar, but treats missing values of NO2 or NH4 as zeros – this increases the sample size of DIN0 relative to DIN, with the assumption that nitrate dominates the DIN composition.

**Fig. 4:**
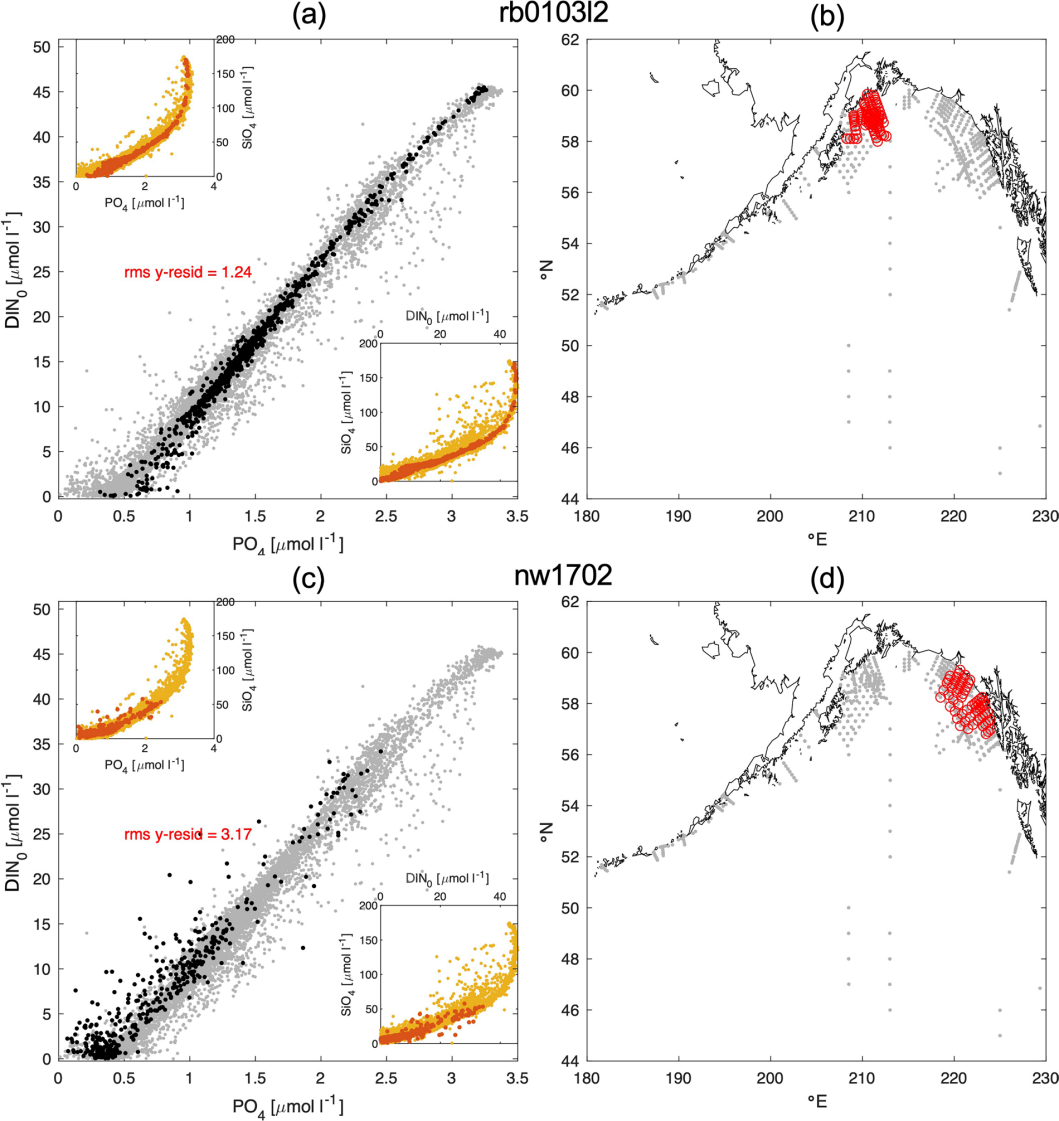
Example property-property plots in the Gulf of Alaska (GOA) for cruises rb0103l2 (top) and nw1702 (bottom) that were used as part of the contextual quality assessment to flag the nutrient data. The primary axis in (**a**) and (**c**) shows the nitrogen-phosphate relationship, where nitrogen is represented by DIN0 as described in the text. Insets show the silicate-phosphate (top left) and silicate-nitrogen (bottom right). Data for each cruise (black or red) are shown against a background of all data in the GOA (grey or yellow). The relationships in (**a**) were typical for offshore data in the GOA, and the scatter in (**c**) was found to be unacceptable for inclusion in the compendium. The station locations for each cruise are shown in red in (**b**) and (**d**) with the grey background showing the location of all stations in the GOA prior to data flagging.

The archival data did not include quality flags for nutrients, and the quality of each analytical measurement was unknown and assumed to be good. For this Level 3 product, we conducted a “contextual” quality assessment of the nutrient data and assigned quality flags. This assessment included regional and cruise-by-cruise property-property plots (Fig. 4), T-S plots, and depth profiles (not shown) to identify obvious instances of spurious cruises and data (e.g., cruises with questionable calibration standards, inverted depths, obvious outliers). Identifying spurious data was easiest in the GOA where offshore nutrient properties are tightly correlated; it became progressively more difficult farther north due to the variability associated with nutrient cycling (e.g., denitrification) and interleaving of different water types on the Bering and Chukchi shelves. As expected, nutrient relationships were also highly variable in nearshore waters. To prevent over-flagging of the data set, only the most egregious data errors were flagged. In several instances entire cruises were removed from the nutrient data set, for example, nw1702 shown in Fig. 4c. Notes and comments made during the quality assessment process were recorded in the string variable “NUT_COMMENT”. The flag assignments were a subset of the WOCE flagging convention^63^, and a separate parameter was provided for each nutrient flag (e.g., “NO3_QC”). Flags are 2 = “good”, 3 = “questionable”, 4 = “bad”, and 5 = “not reported”.

The nutrient data sets also include temperature (“Temperature”) and salinity (“Salinity”) that were taken from the corresponding CTD profile in File **(1)** at the appropriate pressure. Because nutrient analysis began in 2001, all of the corresponding salinities were sampled on the PSS-78 scale, and were drawn from the variable “Salinity_PSS_with_QC_applied” in File (**1**) which is described in more detail below. Note that temperature and salinity were recorded during the downcast of the CTD which provides the highest quality data, while the nutrient samples were collected at discrete depths during the upcast.

Additional derived variables are intended to help users more readily identify whether samples were collected within the upper mixed layer or below. For each nutrient sample, the corresponding CTD data (from Files **(1)** and (**2)** above**)** were used to estimate the profile’s density-based surface mixed layer depth (“MLD10”) in dbar. This quantity is defined as the first pressure level at which the density (sigma-t, kg m^−3^) exceeds the density at a reference depth of 10 dbar by ≥ 0.03 kg m^−3^. Profiles with a missing reference density correspondingly have a missing value for MLD10. Profiles with a valid reference density, but for which no point meeting the MLD criterion could be found (i.e., when the profile is apparently well-mixed within the depth range that was sampled) are listed as MLD10 = −1. An estimate of the isothermal layer depth (“ILD10”) was similarly constructed using a temperature change of >0.2 °C or <−0.2 °C from the reference depth of 10 dbar. For added information on water column structure, the differences in temperature between the sampling depth and 10 dbar is included (“Delta_T10”), similarly for density (“Delta_Sigt10”).

### Reference table

File **(5)** is an additional cruise summary table that includes vessel information and metadata for each cruise. This table lists the cruise identifier (“CRUISE_NAME”) and (“CRUISE_NUMBER”) values, both of which can be cross-referenced to the same variables in the CTD and nutrient files. Additional information includes: the vessel used; number of casts; number of nutrient samples; the date of first and last cast; and longitude/latitude bounds. The “Program” column includes information about the program or project responsible for the main objective of the cruise, where available. Where a cruise can be found in the NCEI World Ocean Database, the NCEI Accession Number(s) corresponding to the data and link(s) containing original archived data are also included. Where possible, accessions that included ACOD variables (CTD and nutrient data) are reported, though in some instances only accessions containing other parameters (e.g., zooplankton data) could be found. These links are included for completeness. In some cases, data were archived in other repositories appropriate for an externally-funded research program (NPRB Integrated Ecosystem Research Programs; GLOBEC), and DOI links are provided for these cruises. Three additional metrics relevant to the salinity QC procedure are also included and are described below.

## Technical Validation

Many of the compendium data are more recent and therefore have been subject to consistent, systematic institutional procedures that are known and documented by the current scientific and engineering staff at PMEL and AFSC. Nonetheless, procedures evolved over time. The assembly of a compendium this size is an opportunity to perform further basic quality assessment, in particular for data from older eras in which the documentation relevant to their processing is not as readily available. Therefore, data included in this compendium were subject to the following additional quality control procedures (beyond typical cruise quality checks described above).

### Initial inspection

The compendium was checked for duplicate data, and a few occurrences were found and corrected. For instance, duplicate data were found within three different cruises (tn179 legs 1 and 3, and ae1001) and the duplicates were removed from the compendium. At times, EcoFOCI, AFSC, and academic partners used different names for the same cruise; these were checked and resolved when assembling archival data. Users of the ACOD data product should take note of these inconsistent cruise names when merging this compendium into larger data sets. For example, a 2009 cruise on the R/V *Knorr* is referred to as kn195, kn195-10, 6N195-10, and 6N195J in various repositories.

**Fig. 5:**
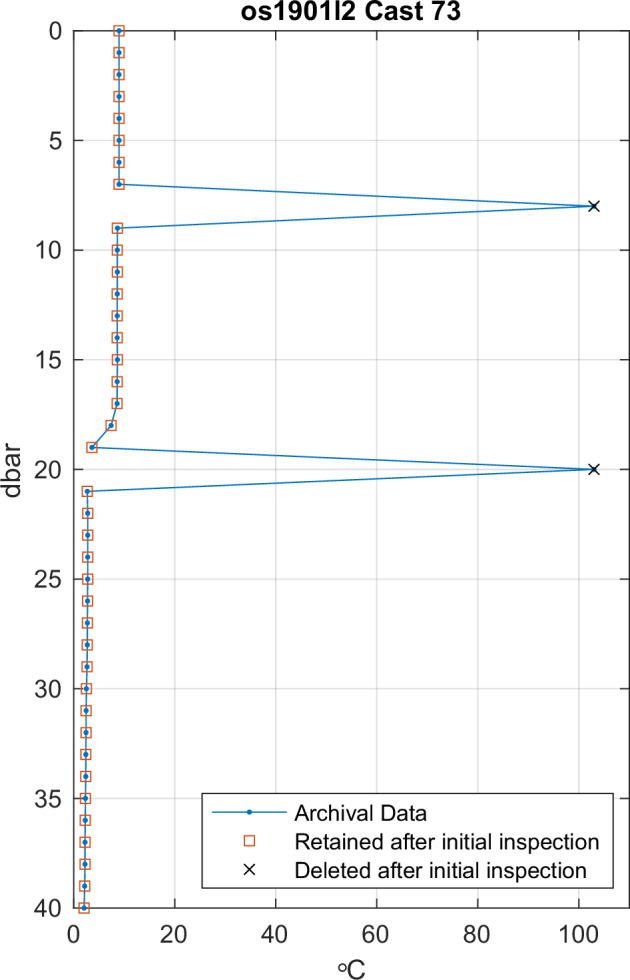
Example plot showing two outliers in the temperature profile of cast 73 on cruise os1901l2 that were identified through visual inspection and deleted from the compendium.

After concatenating cast position and time information from internal EcoFOCI and EMA archives, a basic initial inspection was performed to examine obvious outliers. Temperature and salinity data were visually inspected for outliers using a stepwise approach. Large scale T-S plots (regional or multiple-cruise composites) were used to identify outlier data, with subsequent plots focusing on single casts or groups of casts containing outliers. An example cast containing outlier data for temperature is shown in Fig. 5. This cast contains two points that are outside of physical plausibility, which were deleted in the initial inspection. This procedure resulted in the deletion of some salinity data in 85 casts from 24 cruises, and some temperature data in 12 casts from 11 cruises. The visual inspection of individual cruises identified more systematic errors in salinity in older data, which were addressed using an objective criterion based on static stability that is discussed below.

Time values were plotted for all casts/cruises to identify values falling outside the appropriate bounds for each cruise (e.g., a cast date listed as months later than all other casts in the same cruise). Where possible, erroneous values were compared to the original CTD logs, which allowed correction of transcription errors for 10 casts in 6 cruises. In the end, a total of 4 casts from 3 cruises were flagged and removed for obvious cast date/time metadata errors that could not be corrected.

Similarly, plotting all cast locations in a point cloud relative to the Alaska and Russia coastlines helped to identify obvious location outliers. In 3 casts from 3 cruises, obvious errors could be corrected by comparison to cruise logs. A total of 19 casts from 10 cruises were identified and removed for obvious errors (e.g., latitude/longitude values of zero) when there was no available correction. This inspection did not identify more subtle (small) latitude, longitude, or time metadata errors. Those errors were systematically investigated using stricter criteria discussed below.

### Location and time metadata screening based on implied vessel speed

The location and time metadata were used to estimate vessel speed for quality-control of metadata on a cruise-by-cruise basis. All casts within a cruise were sorted by time, and the minimum distance between consecutive casts was calculated based on the current version of the World Geodetic System ellipsoid (WGS84) and using the *m_idist* function in the M_Map MATLAB toolbox^64^. The listed time between casts was then used to calculate the implied average vessel speed between casts. Cast locations and implied speeds were plotted for each cruise, and transit legs with a vessel speed exceeding 30 km hr^−1^ (16.2 kt) were flagged. This threshold was chosen as a lowest threshold that likely exceeds the maximum speed for nearly all research vessels in the dataset (e.g., for NOAA Ship *Oscar Dyson* see Bahtiarian and Fischer^65^, p. 230), and exceeds typical operating speeds^66^. Thus, a transit leg between two casts that exceeds this speed indicates a likely error in space and/or time coordinates for one or both casts.

**Fig. 6:**
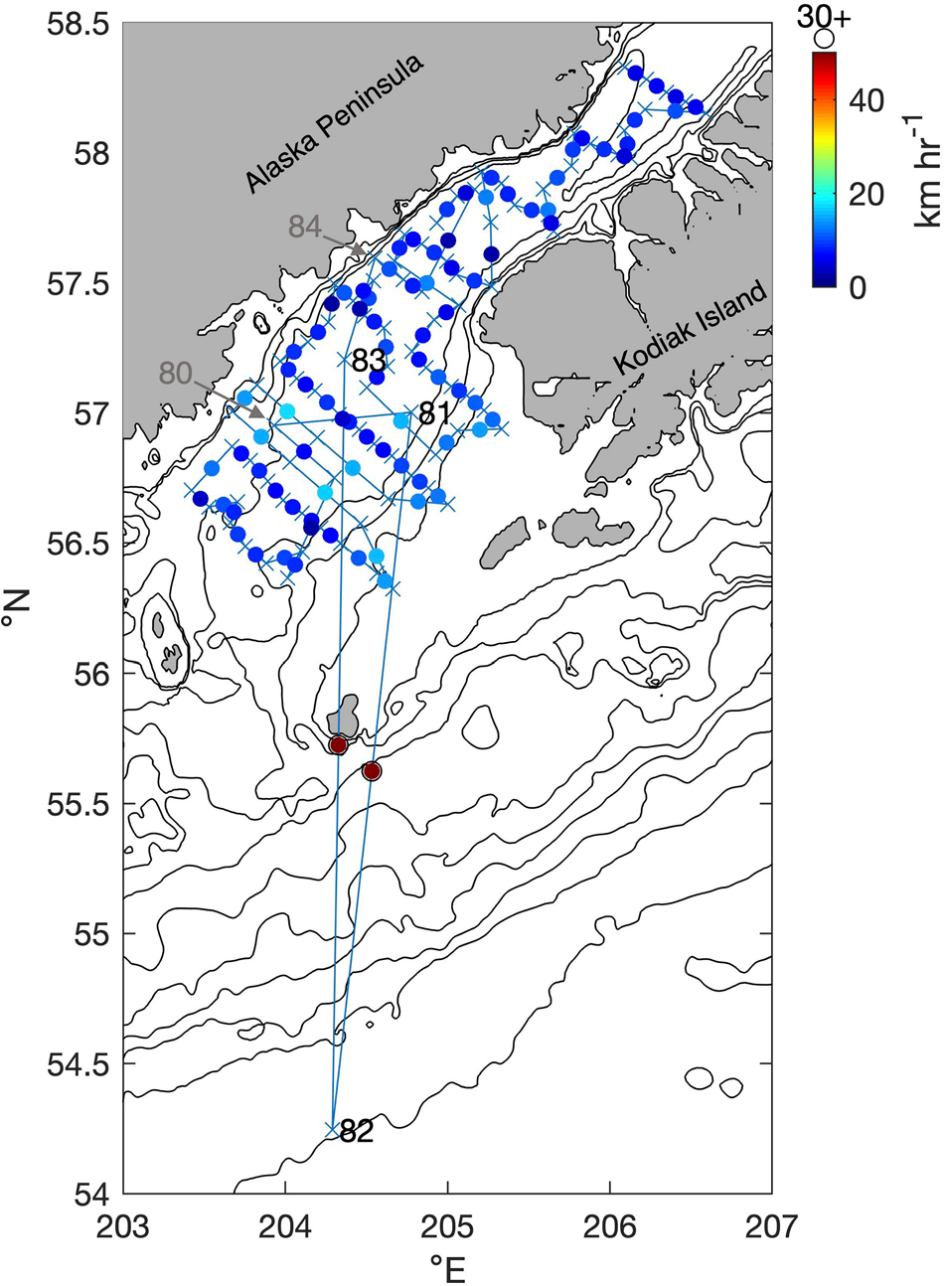
Example plot of cruise mf813b used for examining cast time and location based on implied vessel speed. Blue crosses show CTD cast locations, while blue lines show straight line routes for transit legs connecting adjacent casts in time. The colored circles between each cast indicate each leg’s implied minimum speed, using the scale on the right. Labels indicate the bounding cast numbers for any such legs.

An example spatial plot for a single cruise with >30 km hr^−1^ transit legs highlights an instance of a location error (Fig. 6). This figure shows data (30 March - 8 April 1981) from cruise mf813b (NOAA Ship *Miller Freeman*) in Shelikof Strait and the northern GOA continental shelf. The transit legs between casts 81–82 and 82–83 have an implied speed >30 km hr^−1^ prompting an inspection that found cast 82 was located more than 300 km to the south from its neighboring casts, despite being collected less than 6 hours apart. The most reasonable explanation for this error is an inaccurate latitude value for cast 82. Because CTD log sheets for this cruise were not available for possible correction, cast 82 for mf813b was removed from the compendium. Once deleted, the implied speed for the transit leg between casts 81 and 83 fell within the normal range for this cruise (data not shown).

**Fig. 7:**
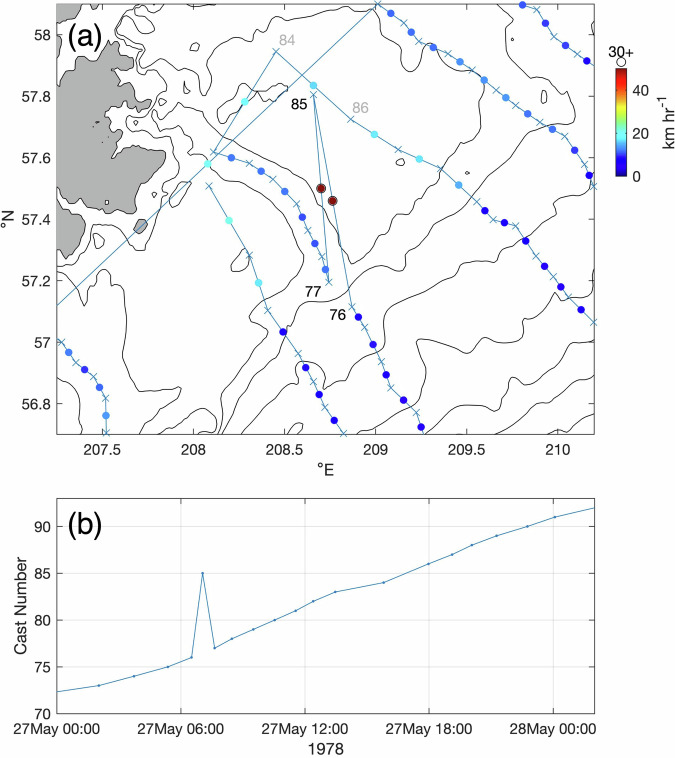
Example of location/time metadata quality control for cruise di785a. The time for cast 85 is listed between casts 76 and 77. Panel (**a**) shows cast locations (blue crosses) and implied minimum vessel speed (colored circles) as in Fig. 4. Panel (**b**) shows cast number versus time for this cruise.

The implied vessel speed was also used to identify likely instances of time recording errors. For example, cruise di785a (NOAA Ship *Discoverer*, 26 May-7 June 1978) east of Kodiak Island, Alaska had an implied speed of >30 km hr^−1^ between cast 85 and the surrounding casts (Fig. 7a). After sorting by time, cast 85 fell between casts 76 and 77 (Fig. 7b) rather than casts 84 and 86. The most likely cause of this discrepancy was a listed time that was approximately 10 hours too early. On occasion, a cast taken after 10:00 UTC may be accidentally recorded without the leading “1” (i.e., 17:03 recorded as 7:03 in this instance). CTD log sheets were not available for this cruise, but correcting the time for cast 85 in this manner resulted in normal transit speeds between all stations. Other instances of 10-hour errors were also corrected. Similarly, casts taken just after 00:00 UTC, but in which the date digit was accidentally not advanced by 1 (resulting in a 24-hour error in cast time), were also corrected.

All cruises were inspected for position and time errors. There were 332 instances spread over 125 cruises that had implied vessel speeds >30 km hr^−1^. Locations and times were cross-referenced with the EcoFOCI cruise log records, and transcription or time recording errors were corrected in 166 instances. There were 160 casts in 82 cruises that had incorrect location and time metadata that could not be corrected, and these were deleted from the compendium. In four cruises (di768a, mw762a, sa813a, and ss777b; between 1976 and 1981), latitude, longitude, and time errors in cast metadata were so widespread that they were not included in the compendium.

There were 65 instances in which cast errors were flagged by the speed criterion, but the exact cast responsible for the error was ambiguous, and the error was judged to be subjectively small in comparison to errors described above. These casts were retained in the compendium despite triggering the speed flag. These occurred in, for example, cruises where shelf CTD stations were taken in close succession such that a time error of 1–2 hours resulted in a large difference in implied speed.

### Screening of salinity data based on static stability criterion

Original, archived salinity data are presented as the variables “Salinity_PPT” or “Salinity_PSS”. Due to the widespread appearance of salinity errors in data sets prior to 1987, additional quality-controlled salinity variables were constructed: “Salinity_PPT_with_QC_applied” or “Salinity_PSS_with_QC_applied”. In these variables, vertical regions of obvious salinity errors in profiles were deleted. The intent was to provide users with a salinity variable that significantly reduced the number of obvious outliers.

**Fig. 8:**
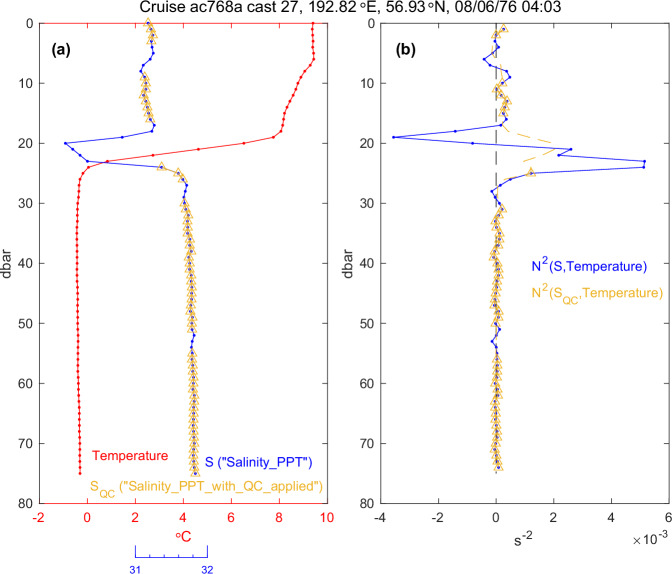
(**a**) Temperature (red) and archived salinity (blue, axes below) versus pressure from a profile taken on the Bering Sea shelf in August 1976. Salinity with suspect data removed (S_QC_, yellow) are also shown. Salinity was reported in units of parts per thousand in this cruise. (**b**) Square buoyancy frequency N^2^ as estimated using the temperature and original salinity (blue), and with salinity that has been linearly interpolated through the removed regions in S_QC_ (yellow, interpolated portions are dashed). Here S_QC_ refers to the parameter Salinity_PPT_with_QC_applied.

The typical mode of salinity error that was observed within profiles was a limited vertical region of positive or negative excursion of salinity associated with thermal stratification, for example, near the transition layer at the base of the surface mixed layer. An example of this phenomenon is shown in Fig. 8a, which displays a profile from the southeast Bering Sea middle shelf in August of 1976 aboard the R/V *Acona*^47^. In this instance, the error is a lower-than-expected salinity value while lowering the instrument. This type of error would be produced by a longer temperature sensor lag compared to conductivity that is consistent with the characteristics of the Plessey instruments that were used aboard this cruise^47^ (Fig. 2 of Roden and Irish^67^). Errors of this type could also be produced by time misalignment between temperature and salinity sensors in the data logging equipment used at that time^67^. In general, a salinity error of sufficient magnitude produces a region within the profile of apparent positive density stratification that is in contradiction to a statically stable profile. In this example, the error occurs on the upper half of the salinity excursion (Fig. 8b). The screening procedure was therefore based on water column stability as measured by the square buoyancy frequency N^2^ (units of s^−2^)

1 $${{\rm{N}}}^{2}={\rm{g}}\,(\,{-}{{\rm{\rho }}}^{-1}\,\partial {\rm{\rho }}/\partial {\rm{z}}+{\rm{\kappa }}\,\partial {\rm{P}}/\partial {\rm{z}}),$$

where g is gravitational acceleration, ρ is density, z is the vertical coordinate (positive upwards), κ is adiabatic compressibility, and P is pressure (IOC, SCOR, and IAPSO^49^, their eq. 3.10.1).

Because a single salinity excursion produces both positive and negative N^2^ errors, selecting depths with unrealistically negative N^2^ values identifies only one half of an excursion. This was addressed through an iterative approach whereby suspect regions were identified, the stability re-calculated by interpolating through these regions, and the vertical extent of suspect regions progressively refined until a stability profile uniformly met the threshold stability value.

An N^2^ profile was calculated from salinity and temperature at each pressure level^68^, using data from levels immediately shallower and deeper (±1 dbar, or ±5 dbar at depths greater than 1500 dbar). For this procedure, salinity data reported as PPT were treated as equivalent to PSS-78. After estimating the N^2^ profile, depth levels with N^2^ < –1 × 10^−4 ^s^−2^ were identified, along with the adjacent points above and below. Salinity and temperature profiles were then linearly interpolated through the identified points and used to re-estimate N^2^. Interpolating both variables during this routine was found to more consistently identify the vertical extent of salinity excursions. (Note that interpolated temperatures were used for quality control of salinity profiles and were not transcribed to the temperature profile data.) After interpolating, any new points meeting the N^2^ < –1 × 10^−4 ^s^−2^ criterion were added, along with their adjacent points, to the list of depths to be interpolated. The procedure was repeated until the profile had N^2^ ≥ –1 × 10^−4^ s^−2^ everywhere (Fig. 8b).

A threshold of N^2^ = –1 × 10^−4 ^s^−2^ is a conservative value. This threshold was chosen to increase confidence that points identified are truly spurious data, with the tradeoff that some less obvious errors were not identified. As a result, it should be noted that the “Salinity_PSS_with_QC_applied” and “Salinity_PPT_with_QC_applied” variables may contain regions that still have negative static stability, or salinity errors that were of insufficient magnitude to produce a reversal in static stability. Users are encouraged to inspect the salinity data prior to their own use with an understanding of the limitations of data collected in the earlier era.

Most CTD casts are initiated at ~2-3 m depth with the salinity at those depths interpolated to the surface. In some instances, there were very low salinity (∂S/∂z < –1 m^−1^) values observed in the top 1-2 m, and samples meeting this criteria were removed in the automated QC procedure.

**Fig. 9:**
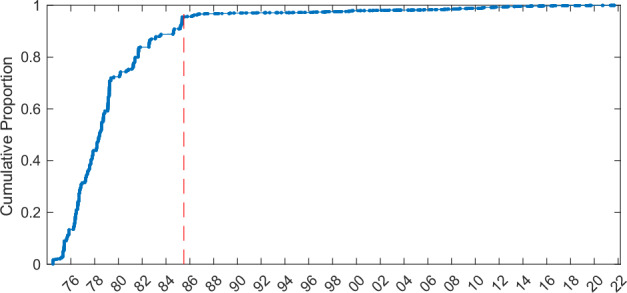
Cumulative proportion of the total vertical extent removed by the salinity QC procedure as a function of date. The red dashed line is 1 July 1985; the first cruise after this time is di857a. Note, the majority of spurious salinity data (95.5%) occurred prior to this date.

**Fig. 10:**
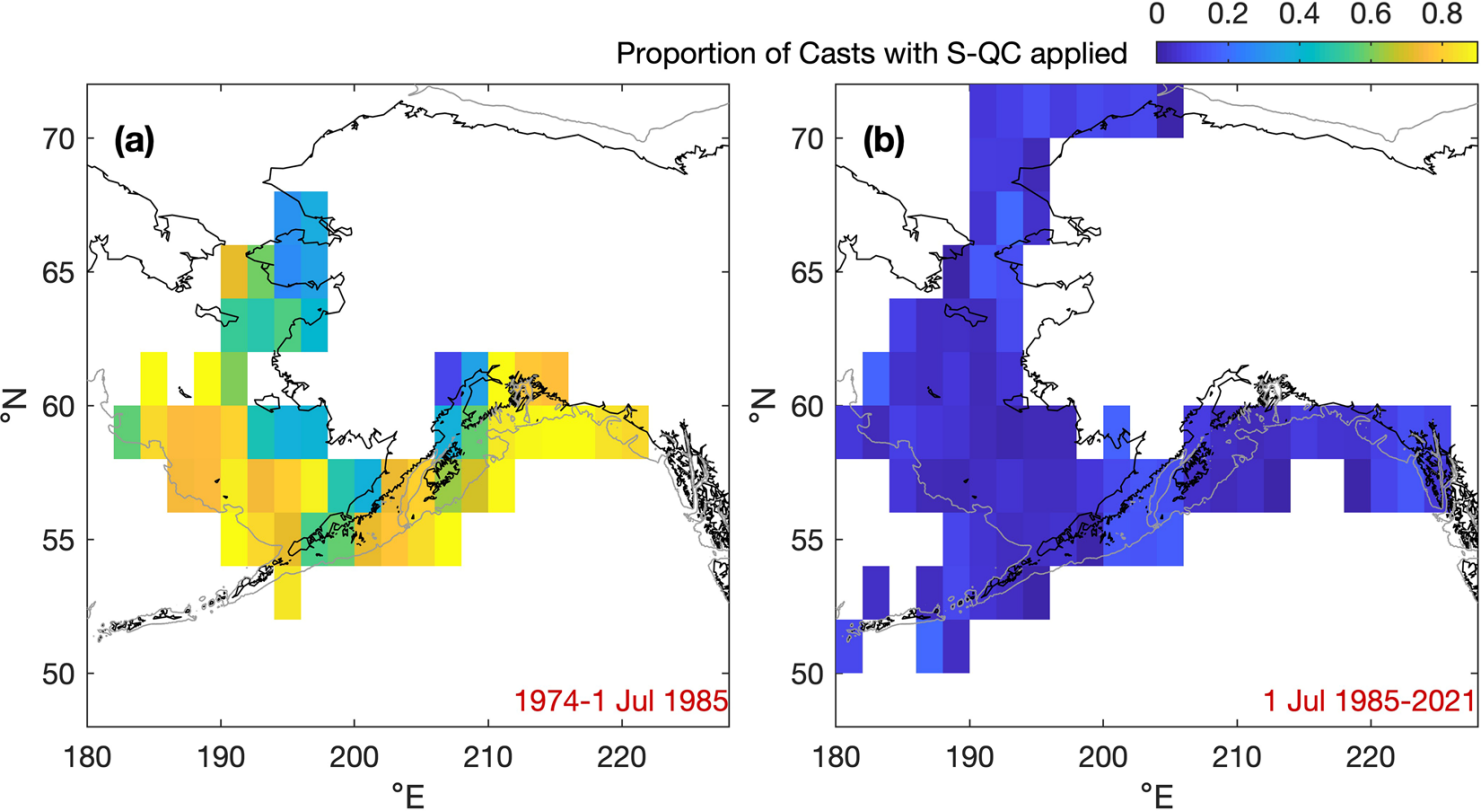
Spatial aggregation of the proportion of salinity profiles in the compendium that meet the criteria for additional salinity QC, before (**a**) and after (**b**) 1 July 1985 (cf. Figure 9). Proportions are estimated in 2° × 2° latitude/longitude bins with ≥ 25 profiles.

The original salinity data have a total vertical extent of 7,464,633 dbar. Of this total, 138,878 dbar was flagged and removed by the salinity QC procedure. The majority of the excluded data (95.5%) were collected prior to 1 July 1985 (Fig. 9. This indicates an approximate temporal boundary in the database after which the quality of the salinity data markedly improved. Prior to July 1985, there were fewer removals on the inner continental shelves (water depth < 50 m), which is weakly stratified or well-mixed, hence less likely to contain thermal stratification leading to salinity processing errors^69–71^ (Fig. 10).

**Fig. 11:**
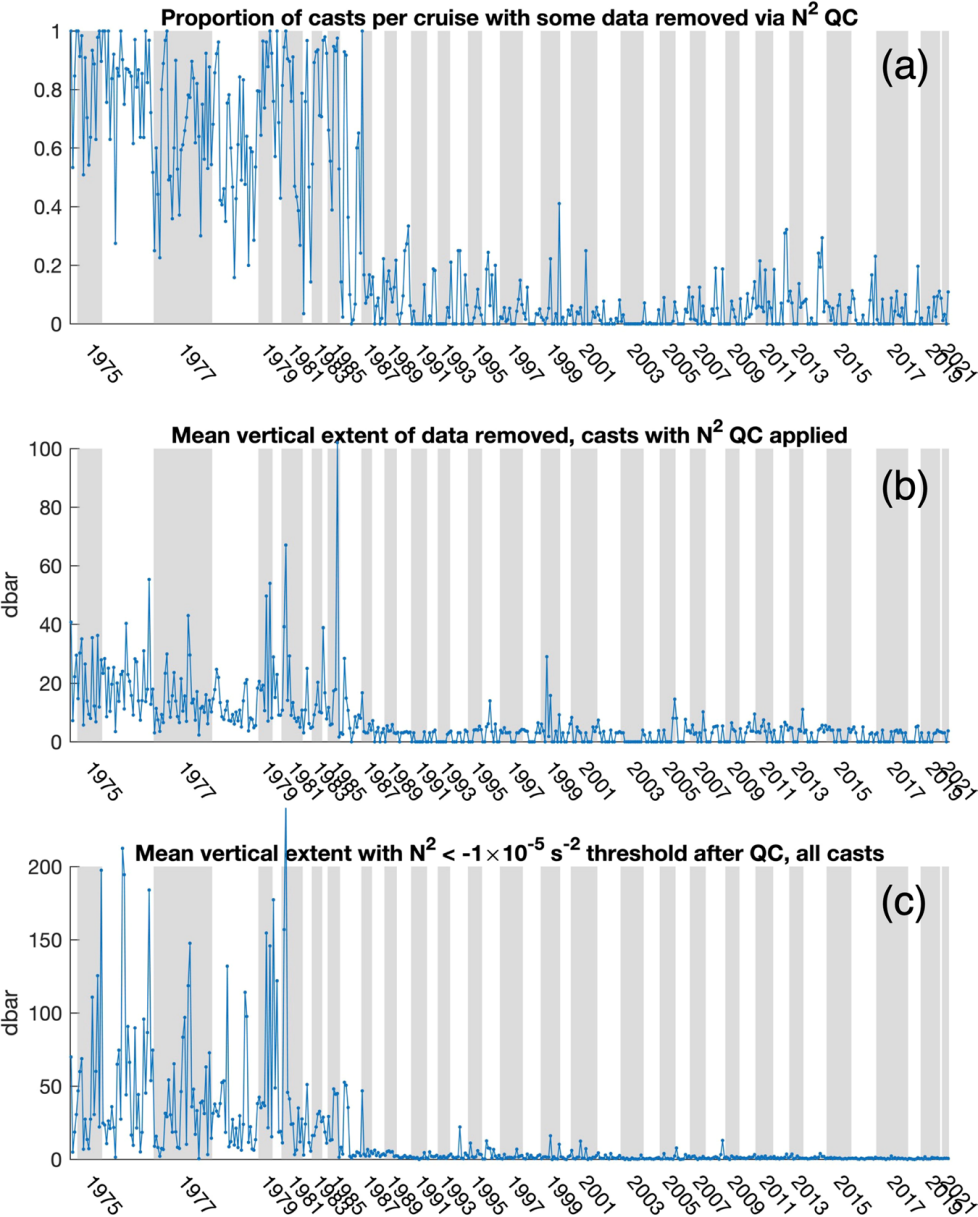
Cruise-average metrics related to salinity quality control (QC). Each data point is one cruise, with cruises ordered by year (background shading). Panel (**a**) shows the proportion of casts per cruise with any data removed by the QC procedure, and (**b**) shows the average vertical extent removed in these casts. Panel (**c**) shows the average vertical extent in all casts with N^2^ < −1 × 10^−5^ s^−2^, where N^2^ has been estimated by linearly interpolating through areas removed by the QC procedure. Note the difference in the y-scale in (**b**) and (**c**).

This era difference in salinity quality is also apparent in cruise-by-cruise plots of the proportion of profiles in which data were removed by the QC procedure (Fig. 11a) and in the average vertical extent removed in these profiles (Fig. 11b). Cruises in 1986 had generally fewer (Fig. 11a) and vertically less extensive (Fig. 11b) removals in the salinity QC procedure, and data definitively improved following the first cruise in 1987 (mf873a). This temporal boundary is also observed when using the average vertical extent that exceeds a lower threshold for instability (N^2^ < –1 × 10^−5^ s^−2^ rather than –1 × 10^−4 ^s^−2^; Fig. 11c) in all profiles. This evidence suggests that the 1985–1987 quality boundary is a sampling boundary, rather than indicative of changes in physical phenomena. These quantitative metrics for each cruise are included in File **(5)** of the data product.

### Quality control of bottom depth information based on profiling depth

Bottom depth data were extracted from the CTD archival cast metadata where available. A total of 1202 casts (~4% of initial data) had missing bottom depth information or had bottom depth listed as zero. Casts with valid bottom depths were screened by comparing the listed bottom depth with the maximum depth of the profile. Sometimes, maximum profile depths exceeded the listed bottom depth by a few meters, especially when profiling over steep bottom topography. However, larger mismatches can be an indicator of cast metadata errors, or an incorrect transcription of the original cruise logs. We inspected bottom depth for agreement with sampling depth, general bottom topography patterns (slope/shelf/passes), and cruise logs. Based on this we took various measures to correct, restore, or delete bottom depth values throughout the dataset where possible (1988-onward for digitized logs).

Errors in transcription were corrected in 7 casts, previously missing values were restored in 36 casts, and values from 170 casts were deleted. Two casts from cruise la0201 in Amukta Pass had bottom depths consistent with known pass depths, but maximum profile depths that exceeded this by hundreds of meters. Because it was not physically possible to have a cast this deep at this location, these two casts were removed from the compendium due to a likely location metadata error. Similar bottom depth alignment inspection led to the removal of 8 additional casts in 2 cruises (nw1201 and mf0802l1).

We retained bottom depths for 62 casts where the sampling depth exceeded the listed bottom depth by > 50 m. These predominantly occurred near steep topography such as Bering Canyon or the GOA continental slope, consistent with expectations as described above. In total, bottom depth is available in 95.9% of the CTD cast data in this initial edition of the compendium.

## Usage Notes

This compendium (ACOD^25^) represents a value-added product for marine research in Alaska waters, bringing together data sets from various archives, and applying additional quality control of data and metadata. It also provides additional simple metrics (e.g., bottom depth, mixed layer depth, geographical region) to augment data selection and ease of use. ACOD is stored in Dryad (10.5061/dryad.gf1vhhn0t), and biennial updates to the archive are planned. Updates will include new data sets generated by EcoFOCI, EMA and other NOAA groups (e.g., AFSC bottom trawl survey), along with continued scrutiny of data quality of existing data sets.

**Fig. 12:**
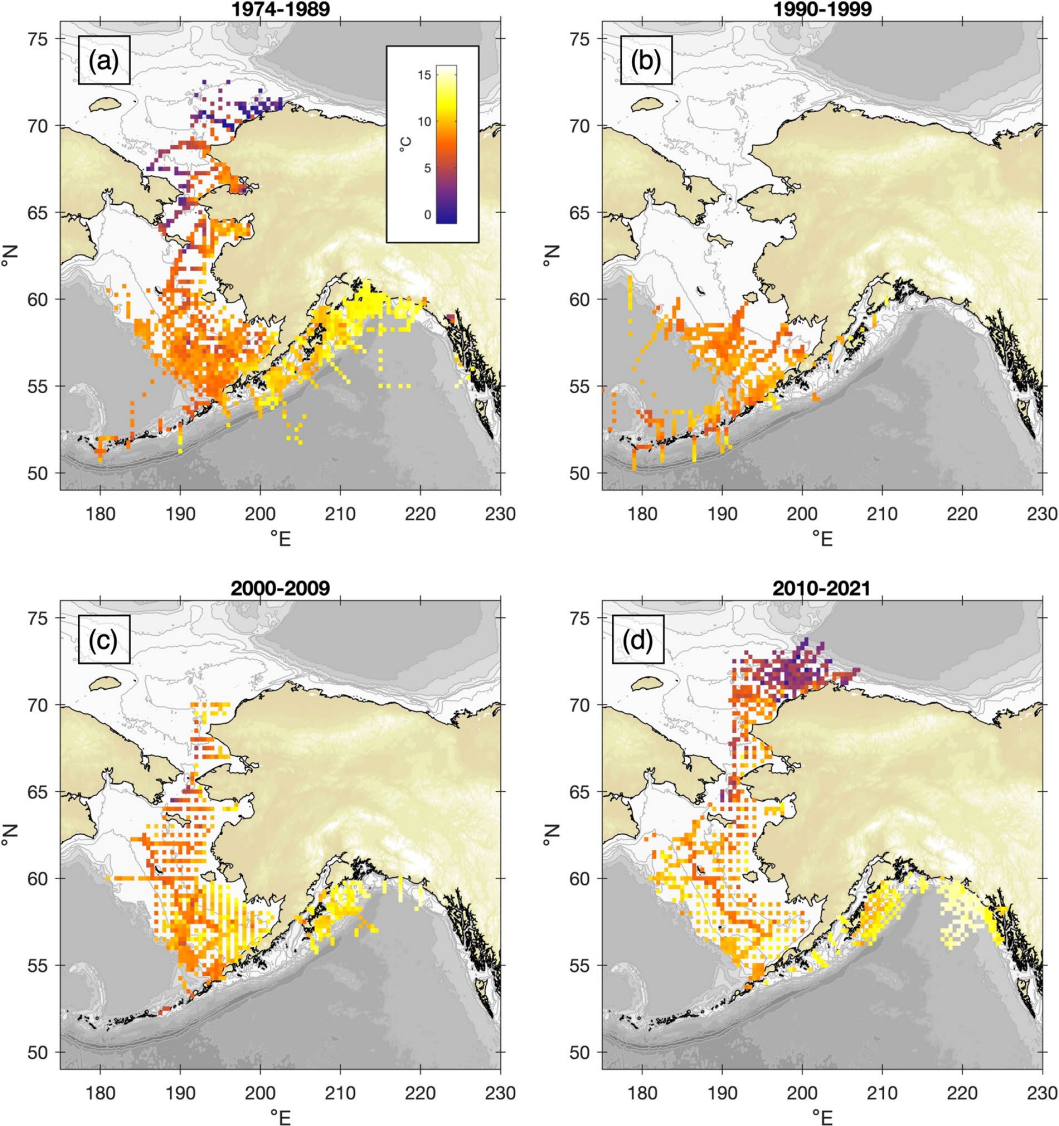
Mean August-September temperatures in the upper 20 m, constructed from ACOD CTD data in the same eras shown in Fig. 1. The colorbar is shown in panel (**a**).

Climatologies of the vertically-averaged upper 20-m ocean temperature in August-September and its decadal evolution (Fig. 12) demonstrate its potential usage in examining spatiotemporal variability of ocean properties across several LMEs. These climatologies were constructed from the ACOD CTD data using the same eras as in Fig. 1, on a 0.25° latitude by 0.5° longitude grid. Data from this product will be useful for multiple studies, including investigations of ocean circulation, vertical structure and water mass properties (temperature, salinity and nutrients), and interannual variability in these rapidly changing marine ecosystems. Earlier compilations of these data have been used in a variety of studies including the seasonal and interannual variability of nutrients in the northern Bering Sea^10^ and the extent of denitrification in the Bering Sea^72^. These data are particularly suited to evaluate the skill of biogeochemical models. For example, data in ACOD have been used to examine interannual variability of water column structure in the northeastern Chukchi Sea^73^, and nitrate data have been used to validate a biogeochemical model of the GOA^74,75^. Ocean hydrographic data incorporated into ACOD have played a critical role in evaluating Modular Ocean Model 6 for the Northeast Pacific (MOM6-NEP) hindcast simulations^76^. Recent studies have used early versions of ACOD to investigate relationships between the physical environment and the EBS shelf ecosystem^77^, and examine the composite structure of the summer nutricline on the EBS shelf^78^.

The dataset is open and available for public use under the Creative Commons CC0 1.0 license (https://creativecommons.org/public-domain/cc0/). If using these data in a publication, please cite this data-description article, in addition to the data record^25^.

## Data Availability

The ACOD dataset^25^ is available on the Dryad open research data repository (10.5061/dryad.gf1vhhn0t).

## Code availability

Custom MATLAB (R2022b) scripts^79^ used to load, inspect, and assemble archival source data into ACOD files can be viewed at 10.6084/m9.figshare.28822796.
